# Aligned Boron Nitride Nanotube Thin Films and Their Cocomposites with Single-Wall Carbon Nanotubes through Slow Vacuum Filtration

**DOI:** 10.1021/acsnanoscienceau.5c00022

**Published:** 2025-06-17

**Authors:** Pavel Shapturenka, Tehseen Adel, Frank M. Abel, Angela R. Hight Walker, Jeffrey A. Fagan

**Affiliations:** † Materials Science and Engineering Division, 10833 National Institute of Standards and Technology, Gaithersburg, Maryland 20899, United States; ‡ Quantum Measurement Division, 10833 National Institute of Standards and Technology, Gaithersburg, Maryland 20899, United States

**Keywords:** boron nitride nanotubes, carbon nanotubes, directed assembly, anisotropic colloids, filtration, global alignment, Raman scattering

## Abstract

Boron nitride nanotubes (BNNTs) are a promising nanomaterial due to their remarkable optical and mechanical properties, chemical robustness, and extended aspect ratios. Herein, we report the formation of strongly biaxially aligned thin films of BNNTs using automated slow vacuum filtration (SVF), as well as their cocomposites with single-wall carbon nanotubes (SWCNTs). Pure BNNT SVF-generated films are found to differ in optimization conditions from those identified previously for SWCNTs but display similar improvements in alignment and uniformity with advanced purification for nanotube length and homogeneity, with globally aligned films observed. Mixed, cocomposite, biaxially aligned films of BNNTs with SWCNTs are also described. Such films provide effective and efficient hosting capabilities for unique morphologies of distributed and individualized SWCNTs aligned by a wide-bandgap BNNT matrix. Concentrations upward of 25% SWCNT mass fraction were found to reside within majority-BNNT films without significantly disrupting the global composite structure; the SWCNT fraction, in turn, enabled probing of both local and global nematic alignment through their use as spectroscopic reporters. Leveraging the thickness and alignment control provided by our SVF implementation, both neat BNNT and composite films show great promise for advancing novel photonic and other thin-film nanocomposite applications requiring tailorable mechanical, thermal, optical, and electronic functionalities.

## Graphical abstract

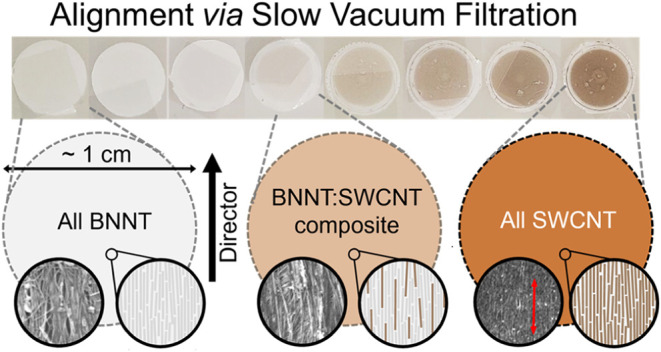

## Introduction

A dielemental homologue of the archetypical carbon nanotube (CNT) structures, boron nitride nanotubes (BNNTs) exhibit their own unique and remarkable thermal, electrical, and optical properties that are promising for technological applications including thermovoltaics, optical resonators and chemical-resistant structures.^1–3^ In particular, BNNT structures similar in diameter to single-wall carbon nanotubes (SWCNTs) or double-wall carbon nanotubes (DWCNTs) are expected to enable unique photonic and electronic functionality through their combination of robust mechanical and wide-bandgap electrical properties. The wide bandgap of BNNTs in particular offers high optical transparency in visible (vis) wavelengths and electrically insulating behavior for applications,^1,4,5^ but results in reduced dimensionality relative to devices demonstrated with hexagonal boron nitride (h-BN).^6^

The native anisotropy of BNNTs naturally drives strong interest for controlled assembly with a global uniaxial (one director axis), biaxial (alignment in a plane toward a director axis), or otherwise structured alignments. Liquid-phase processing methods for attaining such structures are particularly desirable, however, directed alignment via such methods is still challenging, with numerous aspects for controlling the assembly behavior of BNNTs remaining poorly understood. Notable advances include microscopic alignment observed by Kode et al. in DNA-stabilized BNNT solutions;^7^ significant degrees of BNNT alignment in cm-scale thin films by Simonsen Ginestra et al. through shearing acid-stabilized BNNT dispersions;^8^ and achievement of uniform, vertically aligned BNNT arrays and macroscopic structures by Acauan et al. using templated growth on CNT forests.^9^

To this landscape of technologies, we contribute the formation of biaxially aligned BNNT thin films, and eventually their cocomposites with SWCNTs, through slow vacuum filtration (SVF). While no investigations of SVF-based assembly have been previously reported for BNNTs, in the past decade SVF has been developed into an effective process for realizing high degrees of alignment in dimension-controlled SWCNT thin films at scale.^10^ Although contributing mechanisms are not fully understood,^11–14^ and are beyond the scope of this contribution to address, the similar sizes, aspect ratios, and dispersing agents of BNNTs and SWCNTs imply a fruitful translation of SVF to BNNT aligned film generation. There are critical milestones for accomplishing this goal, however, including adapting of SVF protocols to BNNT filtration, applying lessons about population homogeneity from SWCNT SVF for optimized alignment, and to measure the achieved alignment in produced BNNT films.

Several research groups have successfully demonstrated formation of biaxially aligned SWCNT films via SVF. In practical implementations, SVF has produced films demonstrating large areas (several cm^2^) sufficiently aligned to exhibit highly polarized extinction and photoemission, terahertz transduction, chiroptical phenomena, and utility as a hyperbolic metamaterial.^10,15–19^ In both (BNNT and SWCNT) nanotube types, however, synthesis methods still frequently yield polydisperse diameter and length distributions, and can contain high impurity content and variations in morphology at collection; these factors invariably affect the ordering precision of resulting macroscopic assemblies.^20–22^ Due to this reality, separate research efforts have focused on liquid-phase refinement and characterization of both nanotube nanomaterials, with particular recent emphasis on BNNTs.^7,22–26^ Notably for SWCNTs, improved alignment in SVF has been shown after purification, specifically for greater particle aspect ratios and reduced length heterogeneity.^14,27^ Similarly, greater BNNT refinement was shown to increase liquid-crystalline assembly behavior in superacid suspensions.^25^ In this work, we demonstrate significant global alignment of neat BNNTs achieved through optimized SVF, advancing the material refinement and assembly pipeline to realize ever more functional thin-film assemblies. It is worth repeating that no investigations of SVF-based assembly, let alone detailed effects of nanotube length, composition, and film dimensions, have been previously reported for BNNTs.

Realization of aligned BNNT thin films unlocks a particularly exciting potential to integrate and encapsulate other one-dimensional nanomaterials, such as SWCNTs, as guests within mechanically robust and optically transparent aligned composites. As BNNTs are wide bandgap semiconductors with near-unity transmission of wavelengths from ≈ 210 nm into the mid-infrared (IR), they comprise an ideal inert matrix for aligning optically active materials in the visible or near-IR (NIR), while also providing excellent specific mechanical properties (order of GPa for radial and TPa for axial strengths, respectively^28,29^ and thermal conductivity (tens to hundreds of W/m•K).^2^ Although hosting of guest molecules and/or nanoparticles could be endohedral, *e.g*., dye molecules hosted within the BNNT interior, as lattice replacements, or through interstitial templating,^30–33^ a particular motivation for our efforts is in realizing coaligned composites of SWCNTs within a BNNT matrix as illustrated by the center representational image in Figure 1a. Such composites are intriguing for optical cavity and single photon emission applications because intra-SWCNT interactions lead to electronic smearing and depopulation of direct-gap luminescent excitonic transitions in purely SWCNT films. Controlled dilution and templating of isolated SWCNTs within a passive BNNT matrix is expected to reduce such effects, hypothetically leading to increased emission intensity and reduced emission line widths. The availability of thin, rigid, and highly transparent films containing aligned guest SWCNTs should also ease feasibility of mechanical placement and device integration for photonic applications. The contributions of this effort provide the first implementations of such composites, demonstrating the effective templating of SWCNTs over a significant range of loadings. Characterization development to determine the alignment of both neat and cocomposite films is addressed next.

**Figure 1:**
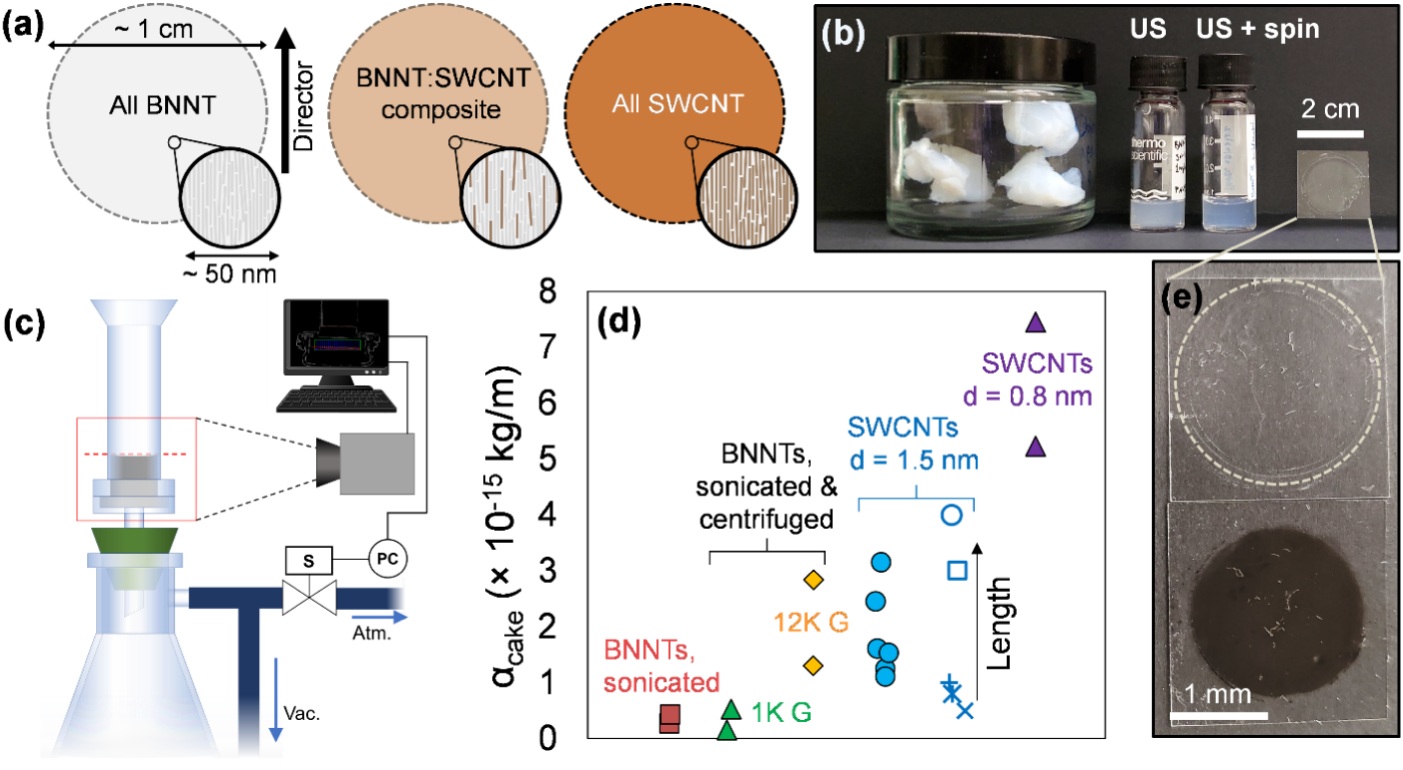
Realization of nanotube thin films through aqueous-phase dispersion, refinement, and analytical slow vacuum filtration (SVF) metrology. (a) Schematic representation of envisioned aligned BNNT/SWCNT composites spanning the entire compositional range from a purely BNNT film (left) to a purely SWCNT film (right). (b) Photograph of BNNT materials used at different stages of processing, from synthesized nanotubes in “puffball” form, to sonicated (US) aqueous dispersions with and without applied centrifugation (spin), to a SVF-filtered thin film. (c) Schematic representation of an automated SVF setup, wherein machine vision monitors the fluid meniscus, and programmed pressure ramping allows flow rate control. (d) Specific cake resistances (α_cake_) of several SWCNT types and BNNTs, including effects of variation in degree of refinement via centrifugation and length sorting. Greater BNNT refinement increases α_cake_ into the range of structurally pristine, individualized SWCNTs, wherein smaller-diameter SWCNTs exhibited the largest determined values. (e) Close-up of the BNNT film (on a glass coverslip), showing the high degree of transparency in an ≈100 nm film, with a SWCNT film of equivalent thickness for comparison. The background is black construction paper.

A challenging consequence of the wide bandgap and optical transparency of BNNTs is that it is difficult to characterize the microstructure of their macroscopic assemblies by common quantitative spectroscopic methods. SWCNTs, in contrast, are readily interrogated with a variety of spectroscopies (e.g., Raman, UV/vis/NIR. linear dichroism (LD), terahertz) by probing their intense, polarization-dependent, and spectrally precise optical transitions. The strongly varying signal allows calculation of commonly described alignment metrics such as the 2D nematic orientational order parameter (S_2D_), capturing the angular distribution of individual nanotubes around a common director, with high precision.^34,35^ Accordingly, we were intrigued by and demonstrate incorporation of SWCNTs templated within an aligned BNNT matrix for use as local spectroscopically active reporters. Such characterization is captured here through polarized micro-Raman spectroscopy on the sub-10 μm to millimeter scale and LD on the ≈ 20 mm^2^ scale, providing a dramatic improvement in meso- and macroscale BNNT alignment measurements. Moreover, evaluation of the thin-film structure over multiple length scales by these methods reveals the effects of smaller domains on the larger scales of observed global alignments and is a metrological advance on its own merits.

## Results and Discussion

The production of aligned BNNT thin films and SWCNT-containing cocomposites required a series of interdependent investigations and technical milestones. We first extend and demonstrate general nanotube alignment via SVF to a parent BNNT dispersion (and SWCNT batches) used in this contribution and identify material-specific properties and system-specific parameters that enable flow rate-based control and rational optimization. After determining an optimized SVF flow rate for the parent BNNT dispersion, photograph in Figure 1b, we do the same for refined length-separated BNNT subpopulations (sorted from the parent) ranging from 150 to 750 nm in average length; the typical diameters of the BNNTs in these populations are between 2 to 6 nm, although with some scatter and observation of larger diameter nanotubes.^37^ As we previously reported for SWCNTs,^14^ significant improvements to BNNT film morphology and alignment are observed for these separated populations due to both absolute nanotube length and length homogeneity effects. Lastly, the potential for templating SWCNT alignment within BNNT films is explored as a function of the guest (SWCNT) concentration, and we present metrological advancements in using such SWCNTs as reporters for probing alignment of the composite films.

### Considerations and Opportunities in Automated Filtration-Based Nanotube Assembly

Most successful demonstrations of SVF for producing aligned SWCNT thin films share several key points: the use of smooth polycarbonate track-etched membranes, a slow and constant transmembrane fluid flux (≈ (0.5–3) mL/(hr•cm^2^)), and implementation of a final compacting “push” step with an elevated flow rate.,^10,11,13^ Control of these factors are paired with surface treatments to the walls of the filtration vessel to minimize disruptive effects from the end stage of filtration, during which the dispersion-air interface can otherwise cause effects such as meniscus combing as it approaches the film and membrane surface.^10,11^ A schematic of the SVF filtration apparatus is shown as Figure 1c. Arguably, the most limiting and challenging of the aspects needed to control the SVF process is flow control. In addition to maintaining an empirically optimized balance of forces, any flow rate variation or greater prevalence of hydrodynamic instabilities (*e.g.,* from fluctuations in the applied pressure difference) are likely to disrupt the correlated structure of the nanotube network accumulated near the membrane (also referred to as the filtration cake).

Filtration of a particulate dispersion is readily described by Darcy’s Law,^11,13,36^ which relates a transmembrane pressure gradient, ΔP, and time-dependent permeate flux, J­(t), to membrane- and filtrate-specific mechanical flow resistances:

1 $$\frac{1}{J\left(t\right)}=\frac{\mu \left(R_{m}+\alpha_{\mathrm{cake}}\mathrm{Cv}\left(t\right)\right)}{\Delta P}$$

where *μ* is the filtrate viscosity, *R*_m_ is a membrane resistance term, α_cake_ denotes specific cake resistance, *C* is the initial nanoparticle dispersion concentration, and *v*(*t*) is the time-dependent, area-normalized retentate volume. While precisely determining permeate flux is not a necessity for successful SWCNT alignment via SVF, it allows precise determination of α_cake_, which broadly reflects the emergent particle shape- and size-dependent compaction behavior that occurs during filtration. As shown in Walker et al., knowledge of α_cake_ is also key to subsequent arbitrary flux control during an automated SVF run.^11^ Figure 1c schematically depicts our in-house automated filtration apparatus, wherein the filtrate meniscus is monitored with machine vision, and a PID pressure control is programmed to keep the total flow rate of the permeate constant. Knowledge of α_cake_ (Figure 1d) for different nanomaterials makes the pressure drop versus flow rate curve at any time predictable, and thus enables improved alignment in produced films; photographs of typical BNNT and SWCNT films are shown in Figure 1e.

The variation in α_cake_ among nanotube materials has heretofore not been systematically explored. As such, three distinct nanotube populations of varying diameter (BNNTs, large-diameter electric arc-discharge synthesized (EA) SWCNTs, and small-diameter cobalt–molybdenum-catalyst (CoMoCAT) synthesized SWCNTs) were subjected to SVF filtrations at constant applied pressure, fully determining eq 1 and allowing a solution for α_cake_ in each case. Using centrifugation as a coarse method for BNNT refinement (collecting the supernatant fraction), the effect of centrifugation speed was also explored. The specific resistance values obtained are reported in Figure 1d. α_cake_ for coarsely refined BNNTs resided in a range between 10^14^ m/kg and 3 × 10^15^ m/kg, increasing monotonically with greater extents of centrifugation of the sonicated-dispersed material. Purified, rate-zonally ultracentrifuged SWCNTs exhibit greater values, with small-diameter SWCNTs approaching 10^16^ m/kg.

Ultimately, cake resistance quantifies the cumulative degree of flow blockage from the bulk fluid to each membrane pore opening; for populations exhibiting greater shape polydispersity (e.g., the BNNTs), the magnitude of α_cake_ is expected to be more modest, with refinement-related increases stemming from a more uniformly compacted cake mass reducing the prevalence of lesser-resistance flow paths from packing heterogeneity. Note that even our initial BNNTs underwent significant chemical purification by the manufacturer and are of relatively high chemical purity, i.e., BN in the form of BNNT rather than as hBN or in elemental form. We interpret the observed increase in cake resistance for centrifugation-only purified BNNT samples as primarily representing morphological selection by the centrifugation, such as the preferential removal of larger diameter BNNTs or less extended-rod morphology objects (*e.g*., aggregates, large bundles, h-BN flakes). Alternatively, in materials where shape uniformity is already high (such as refined SWCNTs), α_cake_ relates to an effective packing density, implying a dependence on packing arrangement (i.e., random versus aligned) and nanotube diameter. These determinations allow us to control permeate flux to within 10% of a given target (≈ (0.05–0.25) mL/(hr•cm^2^), or ≈ (1 – 5) μL/(min•cm^2^)).

Figure 2a features polarized optical microscopy (sample between crossed polarizers, see Methods) of SVF films produced from the BNNT material with and without refinement by centrifugation as described above. We find that SVF of an aqueous BNNT dispersion, i.e., absent of further processing aside from ultrasonication, yields films exhibiting little to no global order. Instead, we observe a mosaic of elongated, nematically ordered microscale domains, and an average transmitted light intensity that varies insignificantly with sample rotation; these findings are reminiscent of liquid-crystalline tactoids recently reported for BNNT suspensions.^8^ SVF of centrifuged dispersions in comparison (at constant filtered mass, see Methods) yields a substantial increase in global birefringent contrast (light intensity change with sample rotation, described in more detail below) in assembled films, accompanied by larger, more isotropic domain shapes. Evidently, BNNT refinement is associated with joint increases in both mechanical flow resistance and structural correlations within the thin-film nanotube ensembles. Supporting this interpretation, atomic force microscopy (AFM) of the centrifuged BNNT material (differential sedimentation) showed mild narrowing of the length distribution with greater applied spin speed (Figure 2c), and some removal of larger aggregates and non-nanotube objects was discerned (Figure S1). In the next section we extend this observation to include findings of improved alignment for longer length nanotube populations and for those with reduced heterogeneity.

**Figure 2:**
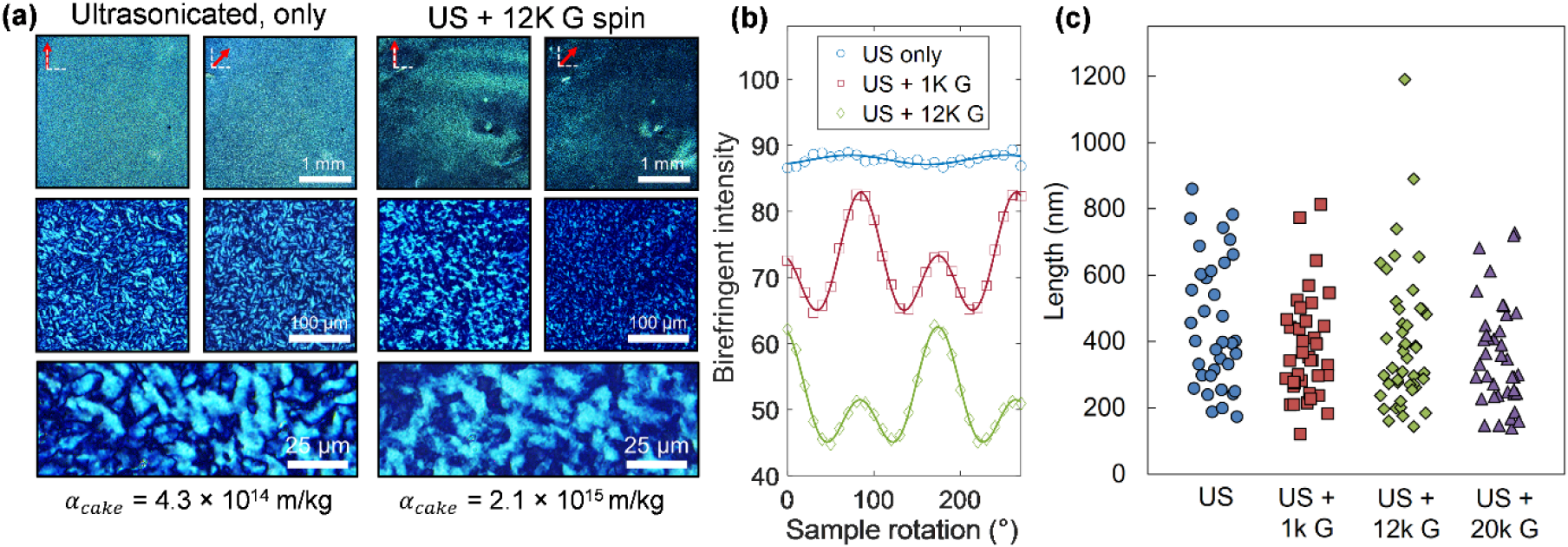
Characterization of thin-film assemblies of boron nitride nanotubes (BNNTs) after various degrees of centrifugation-based refinement, realized via SVF. (a) Cross-polarized optical microscopy images of thin films filtered from two BNNT dispersions: one from an only sonicated dispersion, and the second from its daughter dispersion after centrifugation, with the extent denoted by multiples of the gravitational acceleration constant, G (9.81 m/s^2^) (see Methods for details). Red arrows in the top panels denote the rotation of the films for maximum birefringence (vertical) and at 45° rotation. The film formed from the centrifuged dispersion displays greater global alignment and visually larger domains. (b) Average birefringent intensity of thin films formed from BNNTs with varying extents of centrifugation as a function of film rotation between crossed polarizers. The increasing fractional modulation observed for films from centrifuged dispersions indicates increased anisotropy. (c) Small-sample measurements of nanotube lengths from each processed material population, obtained by AFM, showing a mild narrowing of the length distribution with increasing applied centrifugation.

### Nanotube Length Effects in Aligned BNNT Films under Optimized Flow Control

Prior reports have separately determined that nanotube length and hydrodynamic control are instrumental in achieving global SWCNT alignment. For instance, Rust et al. found optimal alignment at a flux of ≈ (1–1.5) mL/(hr•cm^2^) using a length-narrowed fraction averaging nearly 1 μm in length;^13,14^ Walker et al. arrived at an optimal flux of (1–1.5) mL/(hr•cm^2^) without length sorting;^11^ and He et al. reported an acceptable range of (0.5–1.15) mL/(hr•cm^2^).^10^ Such an optimization window, however, has not yet been established for BNNTs (or other nanotube materials) in SVF. Using SWCNTs as a comparative benchmark, in this section we probe the analogous nanotube length and liquid flux dependence in achieved BNNT assembly. Length separation was achieved using a recently demonstrated for BNNTs polymer depletion-based length separation method (PDLS).^37^ In brief, coarsely refined BNNTs (sonicated and centrifuged at 8 kG, with G ≈ 9.81 m/s^2^) were split into distinct length fractions by iterative steps of precipitation with different concentrations of poly­(methacrylic acid). Five distinct aliquots of length-separated BNNT populations were prepared, denoted in this manuscript as B1–B5, with average lengths of approximately 170 nm (B1) to 750 nm (B5) as determined via AFM and cross-verified with analytical ultracentrifugation.^37^ For comparison and coassembly, arc-discharge SWCNTs were also length fractionated by PDLS in a similar manner,^37^ resulting in fractions labeled A1-A5 and of similar average lengths (see Table S1 for full length details). For each population, the characteristic cake resistance was determined as described above and used to enable SVF of equivalent total masses at a nanotube-material specific constant flux. The resulting films were characterized with polarized optical microscopy; photographs and analysis of the films as a function of the comprising nanotube length fractions are reported in Figure 3.

**Figure 3:**
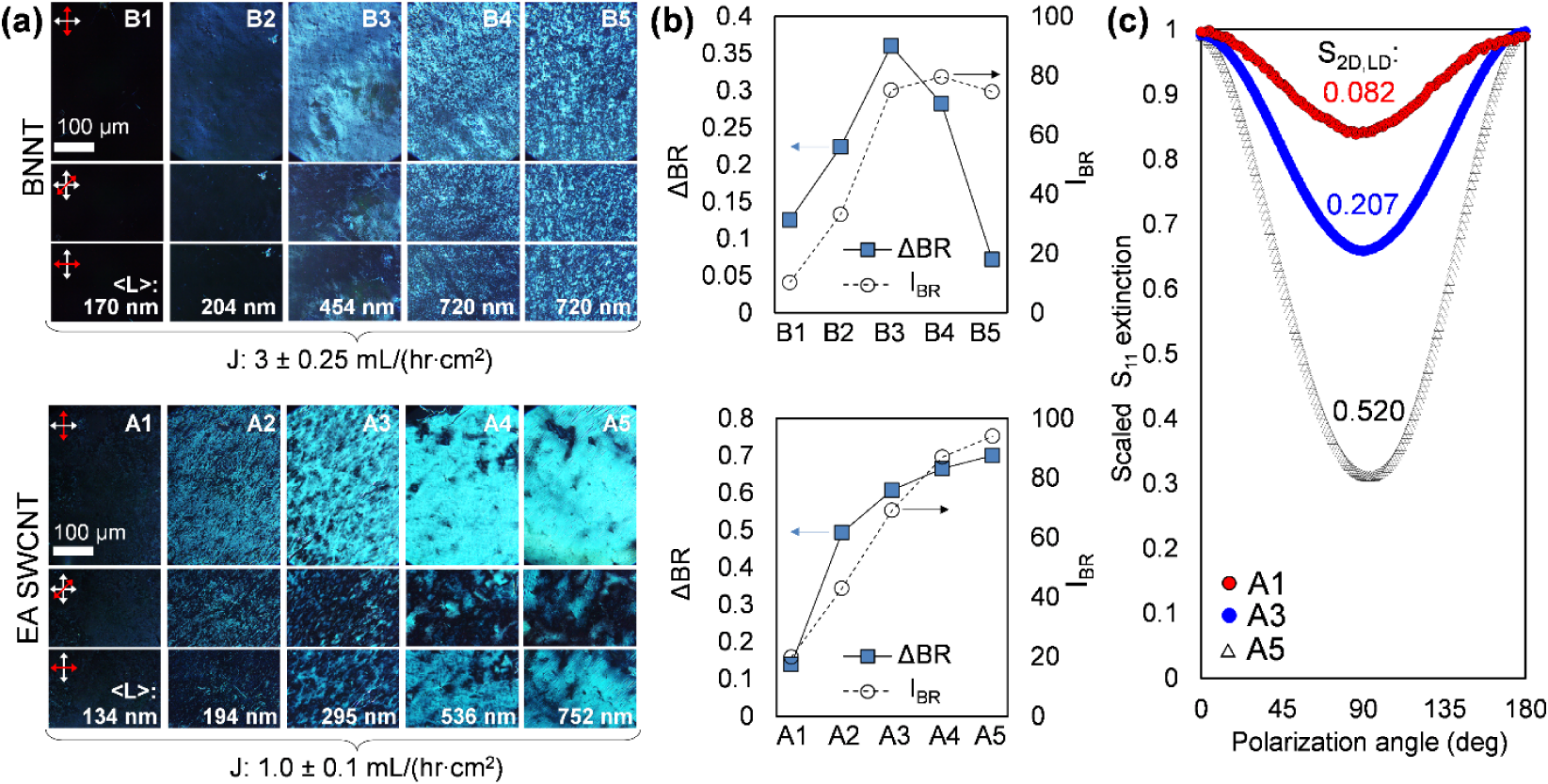
(a) Polarized imaging and spectroscopy of thin nanotube films formed from length-sorted fractions of neat BNNTs (B1 – B5 images) and SWCNTs (A1 – A5 images) at controlled, constant, flow rate conditions after transfer to glass substrates. Average lengths and rotated sample positions are noted in each column and row, respectively. Increased average contrast with rotation implies greater global alignment. (b) Cross-polarized transmission microscopy of length-sorted BNNTs (upper) and SWCNTs (lower), quantified with birefringent contrast (ΔBR), and maximum birefringent intensity (I_BR_), as defined in the main text. Excluding end fractions (1 and 5) to rule out effects of aggregated and non-nanotube material, the greatest degree of alignment at the chosen flow conditions are for B3 and A4. (c) Linear dichroism (LD) of the same length-sorted SWCNT films measured in transmission geometry at the maximum prominent semiconducting species optical transition (S_11_ ≈ 1800 nm) as a function of film rotation in the plane perpendicular to the polarized light source. The films comprised of longer SWCNTs show a dramatic increase in global alignment as demonstrated by the increased variation in absorbance with rotation and the LD-obtained nematic order parameter (S_2D_,_LD_).

In Figure 3a, the representative photographs of the films (comprised of different BNNT and SWCNT length fractions) at various orientations between the crossed polarizers in the optical path contains significant information about the shape and size of aligned domains and the overall degree of alignment in the films. Important considerations for determining alignment within a thin film are, however, both the properties of the aligned material and the measurement method used to probe alignment. Optical methods offer convenient, multiscale, characterization for highly anisotropic objects such as nanotubes and liquid crystals. For BNNTs, their effective transparency in the visible to NIR wavelengths limits the options for ready optical characterization to birefringence (or nonoptical microscopy methods); notably, although BNNTs have an absorbance peak at ≈ 206 nm, the shortest wavelength for LD on a major commercial spectrophotometer is ≈ 300 nm, and capabilities for sub 300 nm LD were not available to our group. Detailed optical characterization information for all the source materials and selected samples is in Figure S2. For SWCNTs, in contrast, the presence of strong intrinsic and anisotropic absorbance features across the UV through NIR wavelengths enable the use of LD and polarized resonance Raman spectroscopy in addition to birefringence measurements utilizing their anisotropy in refractive index.

A versatile method used to rapidly probe nanotube alignment is birefringence, generally measured in a transmission geometry with the sample located between two orthogonal (crossed) linear polarizers. In this configuration, light should only be transmitted if the intervening film causes rotation of the linear polarization state of incident light. Dark films or regions indicate either a low degree of total light rotation due to limited film anisotropy or direct registration of high alignment with one polarizer; these scenarios are distinguishable through rotation of the film. Bright films or regions indicate significant optical rotation of the light by the film, signifying a high degree of nanotube alignment at a given point. To quantify birefringence of such samples we use the measurands I_BR_, which captures the aggregate degree of local nematic order in each nanotube assembly, and ΔBR, which directly reflects the absolute extent of global alignment along a singular director; see Methods for details of I_BR_ and ΔBR calculations. The probed area for the quantified measurements was ≈ 0.2 mm^2^. A more quantitative method to probe absolute alignment is LD, which measures the optical extinction of various light polarizations within the film.

Each of the different optical characterization methods in Figure 3 (I_BR_; ΔBR; LD) provides distinct and complementary support for significant global alignment trends. In I_BR_, both BNNTs and SWCNTs show a monotonic increase and saturation at a maximum intensity for the longest length fraction. This suggests a continuous rise in pairwise orientational correlations (whether within or among domains) with a greater nanotube aspect ratio. Formation of locally aligned domains, much larger than the individual nanotube length, become prominent for shorter length SWCNT fractions than for BNNTs (these characteristic mesoscale domains approach tens of microns in A2 and B3 for 194 and 454 nm average nanotube lengths, respectively). A particularly high degree of alignment was observed over macroscopic areas for the longest separated EA-SWCNTs. This observation was also supported by linear dichroism (LD) measurements (≈ 20 mm^2^ measurement area), which enables extraction of lower bounds for the global alignment (*vide infra*) as quantified by the two-dimensional order parameter (S_2D,LD_) of 0.08 and 0.52 for A1 and A5, respectively, calculated as

2 $$S_{2D,\mathrm{LD}}=\frac{\alpha_{∥}-\alpha_{⊥}}{\alpha_{∥}+\alpha_{⊥}}$$

where α_∥_ is the magnitude of the polarized extinction along the perceived alignment director, and α_⊥_ is the extinction perpendicular to it. Notably, this metric tends to be a lower bound on the true S_2D_, due to nonexcitonic background signal absorbance contributing to the calculation.

BNNT birefringence intensity was maximized in B3 and B4. In particular, the striking uniformity and high contrast of B3 appears to be a novel observation of BNNT alignment with SVF methods. Absolute birefringent contrast also peaked in this region for SWCNTs, but global ordering was more preserved in A5 relative to the same fraction in BNNTs. We attribute this to a disrupting presence of ultralong, aggregated, and defective objects likely present in the longest BNNT fractions as it will sort with long BNNTs. Such objects were likely removed in a rate-zonal ultracentrifugation (RZU) separation that was part of the parent SWCNT dispersion preparation;^38^ RZU processing for extracting structurally pristine BNNTs may be feasible but has yet to be developed as a refinement method for the material.

To sample hydrodynamic effects on the SVF process in a straightforward manner we use fractions A4 and B3. This enables us to explore the dependence of the achieved alignment on the permeate flux with the greatest hypothesized contrast (alignment ability) while neglecting potential confounding effects from precipitated aggregates and non-nanotube material left by the length-separation process (i.e., fraction 5 samples). Polarized microscopy images and quantification of the films are shown as a function of the permeate flux for the BNNT fraction in Figure 4; the results for the A4 SWCNTs are featured in the Figure S3. In line with prior literature, SWCNT assembly behavior was found to be quite sensitive to the flux during SVF, reaching a local birefringence optimum in the range of (1 to 1.5) mL/(hr•cm^2^). Unlike the macroscopically continuous and uniform domain profile observed at the optimal flux, SWCNT film morphologies at the sampled flux extrema resulted in smaller (tens of microns) and more heterogeneous birefringent domains. There were differences in the effects of the extrema in sampled flow rates, however, in that a lesser flux still generated elongated domains retaining some global order, while faster flow rates eventually fully randomized the nematic domain orientation.

**Figure 4:**
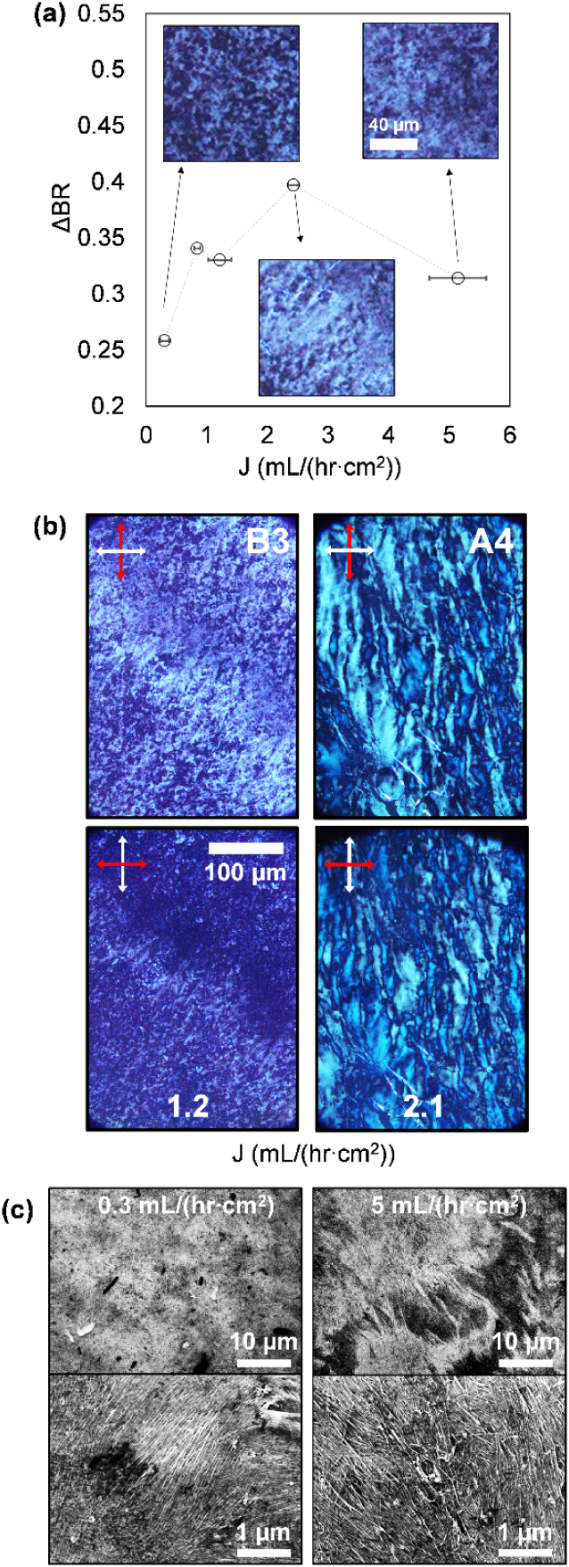
Probing the effects of filtration flux on the multiscale structure of BNNT thin films. (a) Birefringent contrast of BNNT nanotube films formed from the B3 length-sorted BNNT fraction at varying flux, with insets highlighting the structural differences at either end of the studied range. (b) Polarized optical microscopy of nanotube thin films (formed from BNNT/SCWNT fractions B3 and A4, respectively) formed at fluxes optimized for the opposite nanotube type, showing that global ordering and uniformity is viable over a broader flux range in BNNTs relative to SWCNTs. The macroscopic nonuniformity in brightness in the BNNT images stems from film thickness variation. (c) SEM micrographs of the BNNT film surface from two extrema of the studied flux range, showing mesoscopic differences in global BNNT ordering.

In contrast to SWCNTs, birefringence-determined order in BNNT films was found to be dramatically less sensitive to the magnitude of permeate flux (specifically fraction B3). Shown in Figure 4a, the optimal flux was determined from the maximum ΔBR to be ≈ 2.5 mL/(hr•cm^2^), approximately twice that of SWCNTs, but displays a less than 50% reduction from the maximum across the entire measured flux range. While the difference in optimal flux may partially arise from differences in average nanotube length (altering the interaction regime between convection and rotational diffusion), similar trends appear in the observed morphology when comparing to SWCNT films. Fluxes at both ends of the measured range (i.e., above and below the optimum value) yielded smaller, marginally less globally connected domains (Figure 4a insets). SEM imaging (Figure 4b) further elucidates structural differences in the characteristic arrangement of BNNTs; while the film assembled at slow flux appears more uniform overall, the alignment is less prominently global and features smaller, discrete, and misoriented domains, the majority of which happen to align along a significant director as seen by birefringence. Conversely, despite its apparent heterogeneity under SEM, the film assembled at the greatest flux adopts an evidently preferred director that appears continuous at the 100-μm scale in both optical and electron microscopy. Overall, the contrast in flux sensitivity between CNTs and BNNTs is particularly evident when swapping filtration flux for the two nanotube types, as featured in the birefringence images in Figure 4b; the B3 fraction (BNNTs) appears to align readily even at slower flux, while the extended, uniform nematic order observed in the filtered A4 (CNTs) fraction is broken at the relatively elevated fluxes optimized for BNNTs.

The above findings corroborate the length dependence on SWCNT assembly first reported by Rust et al. and more generally extend these phenomena to BNNTs.^14^ In addition to validating earlier work, this is notable because the nanotube length populations used in this work were only separated into 5 fractions rather than the ≈ 12 fractions typical for size exclusion chromatography (SEC).^37,39^ The fractions here thus were of higher polydispersity than those used in Rust et al., although equivalent or finer resolution is achievable via PDLS by applying more stages.^37,40,41^ We posit that the exact breadth of the length distributions is less vital than removal of both length extremes from the parent suspension in the daughter fractions; although often small in mass fraction, those extrema significantly increase the overall polydispersity and have specifically been reported to notably disrupt ordering in other nanotube alignment methods.^27^ Simply removing the tails would also advantageously allow use of a greater portion of total refined nanotube mass in the SVF assembly. To examine this postulate, we assembled SWCNT films by SVF after merely excluding the short length tail of nanotubes from a parent dispersion population; birefringence images comparing these films are reported in Figure S4. Although detailed study beyond the scope of this contribution would be necessary to fully quantify the improvement achieved by solely excluding the short tail, a visible increase in the size and uniformity of aligned domains is observable in the images.

The reason for the exact functional form of the BNNT SVF-induced alignment with increasing values of constant applied flux rate, i.e., why peak alignment is observed at ≈ 2.5 mL/(h cm^2^), remains unclear. However, this is also true of the overall governing mechanism(s) of SVF, for which several hypotheses have been made to explain results obtained under somewhat different conditions. In terms of permeate flux, the magnitude studied herein spans a regime that competes time scales of various phenomena including: (i) downward nanotube advection (≈ microns/second), (ii) diffusion (translational and rotational, order ≈ μm^2^/s and 100 rad^2^/s, respectively,^42^ and (iii) pairwise and collective internanotube interactions (collision, jamming/rigidity transitions). When the permeate flow regime is constant and effectively laminar at all scales (conditions used in this work), the flat cylindrical volume element immediately adjacent to the filtration membrane accumulates mass at a constant rate of continuously descending, randomly distributed nanotubes; although this must naturally increase internanotube collision frequency and precondition the system for jamming, at a constant flux such interactions are most likely consistent. Importantly, irrespective of the mechanism, the effect of flux *variability* on alignment fidelity is likely cumulative and disruptive to the influence of the net director field. Disturbances in flow may cause structural rearrangement in a partially jammed nanotube network to an extent where a globally aligned state may no longer be maintained or reestablished. Here, multiscale computational fluid dynamics studies, microfluidic systems, and percolation theory could be of great use in elucidating and capturing the particulate dynamics giving rise to end-state structural arrangement.^43–45^

In contrast, reasons for improved alignment with increasing nanotube length (L) are more tractable to assign, at least for nanotubes much shorter than their persistence length (persistence lengths are ≈ 10+ μm for the CoMoCat SWCNTs, increase to ≈ 40+ μm for the EA-SWCNTs, and are up to 100+ μm for the BNNTs used in this work). One simple idea is that, for the constant mass films produced here, the number of particles needing to reach proper alignment decreases linearly with L. Moreover, dipole-like interactions are enhanced with L, and there is a greater per-particle penalty to misalignment. Rod-like systems also exhibit concentration-dependent rheological transitions that are highly length-dependent, scaling with L^3^.^3,46,47^ In SVF, this may be particularly important in mediating the extent of structural rearrangement and alignment during cake compaction up to the final fixation of particle positions during the subsequent drying step.

### BNNT–SWCNT Composite Formation and Resulting Opportunities in BNNT Film Metrology

Having demonstrated significant global alignment of BNNTs via SVF, it becomes worthwhile to explore their use and limitations in hosting guest molecules or nanoparticles, particularly in templating the alignment of individualized SWCNTs within such a wide-bandgap BNNT matrix. Although one eventual goal is to disperse and align single species of SWCNTs (defined by the (*n*,*m*) index of their carbon lattice vector and typically called “chirality”) within a BNNT film, to explore the potential for such implementations we use the same nanotube length populations described above. Explicitly, we chose the fractions exhibiting the maximal global alignment while displaying minimal length polydispersity (fractions A4 and B3); the average outer diameter of nanotubes in these samples are ≈ 1.8 nm and 3.9 ± 2 nm, respectively.^37^

To explore the effects of a cocomposite SVF filtration we generated a series of films in which the BN/C mass fraction was varied from 0 to 1. For all films, the loaded nanotube mass was held constant at 8 μg, and SVF was conducted using a constant flux of ≈ 3 mL/(hr•cm^2^). Importantly, this flux targeted the optimal conditions determined above for BNNT assembly, but was performed on mixed together volumes of the A4 and B3 parent dispersions. Our expectation, due to the homogeneous mixing, identical dispersing agent, and similar particle size scales and geometries (and accordingly hydrodynamic interactions), is thus that the SWCNTs and BNNTs should form cocomposites of coaligned structures as visually represented in Figure 1a. All films, photographs of which are shown in Figure 5a, were approximately 30 nm thick.

**Figure 5:**
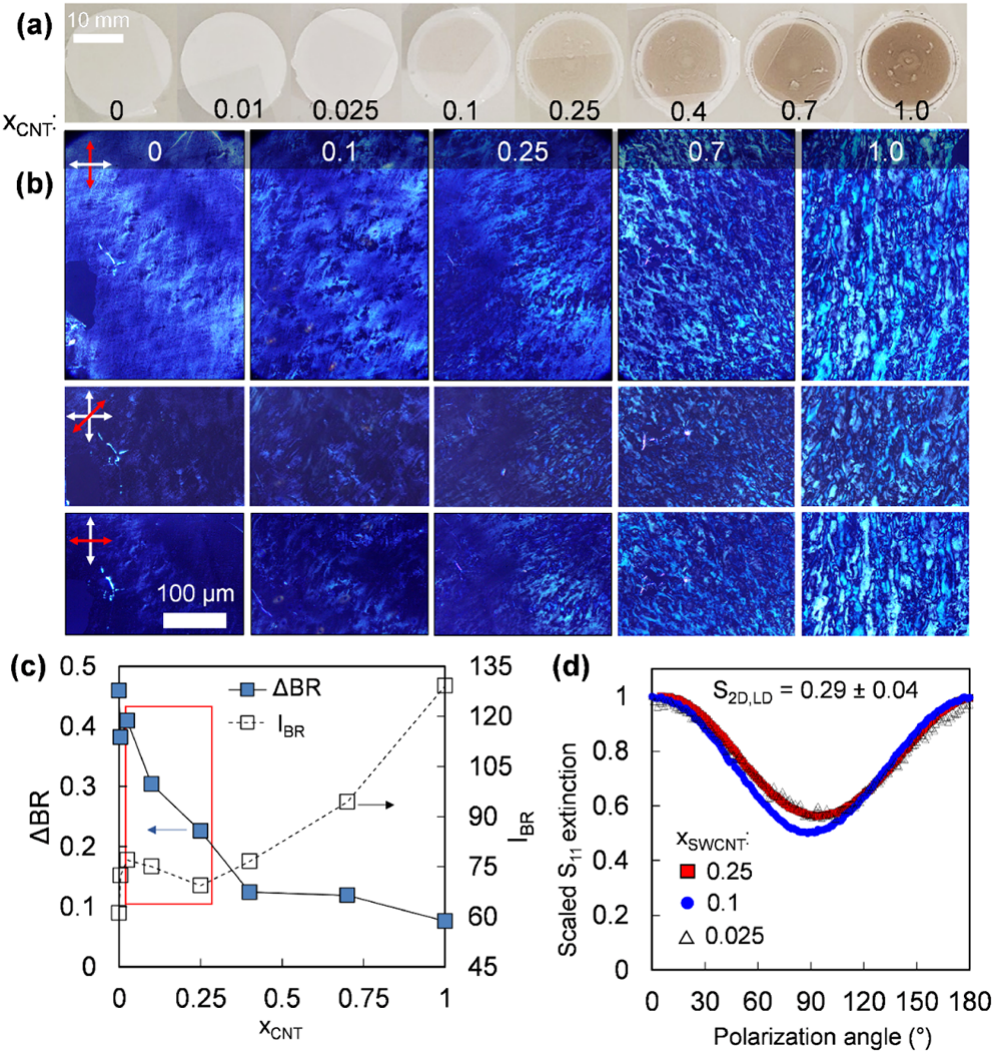
Structural and optical characterization of refined, aligned BNNT/SWCNT composite thin films. (a) Photographs of composite films and (b) cross-polarized microscopy of films transferred to glass substrates. Films display an evolution of domain size, morphology, and orientation with increasing SWCNT mass fraction, x_CNT_. (c) Birefringence measurands as a function of x_CNT_. I_BR_ and ΔBR report a decrease in birefringent contrast while increasing in total birefringence with x_CNT_, the latter is a result of greater SWCNT extinction in the spectral wavelength range of the light source. (d) LD at the maximum excitonic optical transition of the arc-discharge SWCNTs in the NIR embedded in the aligned thin film composites. This value is a macroscopic reporter of the overall global ordering of the SWCNTs as templated by the BNNTs. While cross-polarized microscopy indicates changes in domain morphology across moderate SWCNT compositions, the degree of CNT alignment when measured at a macroscopic length scale is nearly identical.

As described above, changes in domain morphology and alignment of the assembled composites were first characterized with cross-polarized microscopy, with the results shown in Figure 5b. A key finding, seen in the similarity of the 0 and 0.1 mass fraction sample images, is that a small fractional SWCNT content does not appear to affect the morphology or alignment of the dominantly BNNT film. However, introducing over 10% (mass basis) of SWCNTs into the thin film composite begins to dramatically reduce the birefringent contrast, with the decrease leveling off at a CNT fraction of ≈ 40%. Inversely, the total birefringent intensity increased due to the formation of local, ordered domains (remembering that filtration was optimized for BNNTs) with greater visible-wavelength extinction in the latter majority-SWCNT thin films.

While birefringence microscopy is a powerful multiscale method for assessing spatially dependent nematic character, deeper quantitative analysis of the birefringence in the mixed films, in particular, is hampered by the distribution of refractive indexes present, as fundamentally as a material property it has different values and polydispersity for the two materials (e.g., varying with BNNT diameter and SWCNT (n,m)) and additionally dependence on the wavelength (s) of the light source. Moreover, the lack of a simple, well-defined, and invariant optical reference limits its quantitative precision in capturing orientational distributions at all length scales. Unfortunately, unlike SWCNTs for which film alignment can be quantified by LD or polarized Raman spectroscopy as an alternative method, pure BNNT films lack the presence of feasibly accessible optical transitions and thus the possibility of measurement using LD or polarized resonant Raman spectroscopy. However, if we posit that small fractions of SWCNTs are incorporated within a majority BNNT film without affecting the organization, i.e., essentially as tracers as illustrated in Figure 1a, including SWCNTs in the cocomposite SVF-produced films solves this issue and makes additional characterization tractable.

Accordingly, a combination of LD and polarized Raman spectroscopy was used to quantify orientational order for cocomposite SWCNT-BNNT films at different length scale ranges from macro- to meso- to the nanoscale. For the largest scale, LD was used to sample an area of ≈ 20 mm^2^ (5 mm diameter circle). As shown in Figure 5c, despite the prominent differences in birefringent contrast, three films spanning (2.5 – 25) % mass fraction of SWCNTs in the BNNT matrix exhibited nearly identical polarization activity, in each case yielding an estimated lower S_2D_ bound of 0.29, corresponding to an in-plane angular distribution with a standard deviation of ± 42° around its average global director. Surprisingly, the increase in observed heterogeneity in local birefringent domain textures with SWCNT fraction is barely reflected in the overall degree of order in the films. This observation could hypothetically arise from several mechanisms, including phase separation of nanotube materials, differential degrees of responsiveness to the director by the two materials, an improved alignment in larger domains to the global director offsetting the appearance of more domains, *etc*. Neither our birefringence measurements nor LD above are sufficiently quantitative and sensitive at the length scales necessary to be good probes for this mechanism, and phenomena such as compositional nonuniformity become difficult to deconvolve without a precise, spatially resolved, and self-consistent measurement. As such, we turn to another spectroscopy enabled by the presence of SWCNTs in the composite.

Polarized Raman scattering microscopy (PRSM) is a powerful and spatially precise method for quantitatively evaluating the orientation of nanomaterials. SWCNTs lend particularly well to such study due to their very strong optical transition resonance amplification of Raman scattering, and the dominance of the optical modes along the axis of the nanotube; the depolarized scattering intensity from SWCNTs scales with cos^4^ of the separation between the nanotube axis and parallel (V) polarized illumination.^35,48,49^ Here we use PRSM to offer further complementary insight on the fine structural detail of the thin film composites, spatially mapping the local degree of nanotube alignment, and the absolute orientation. Figure 6 shows characterization by PRSM using the Raman scattering (RS) of the SWCNT component inside several BNNT/SWCNT composites. Figure 6a presents a schematic of the light path in the PRSM method and Figure 6b presents an example of RS from a moderately well aligned position in the 10% composite film. The SWCNT subpopulation in resonance at 632.8 nm excitation for this diameter of SWCNT are metallic in nature, leading to a multipeak set of RS features in the G-band region. Due to the anisotropy of SWCNTs, these features are intense for along-axis (0°) illumination and weak for perpendicular (90°) illumination (commonly referred to as the VV and HH positions). The S_2D_ estimate (lower bound), determined using just the VV and HH values shown in Figure 6b from a representative spot is ≈ 0.81. Prior spatially resolved Raman characterization of SWCNT films (as conducted in Walker et al. and Rust et al.^11,14^ measured the local misalignment from a single reference vector using sets of VV, VH, and HH polarizer positions for all points. While effective in evaluating the presence of global unidirectional alignment, that methodology only indirectly classified and evaluated the sample microstructure, limiting further insight and informed iteration on the assembly process.

**Figure 6:**
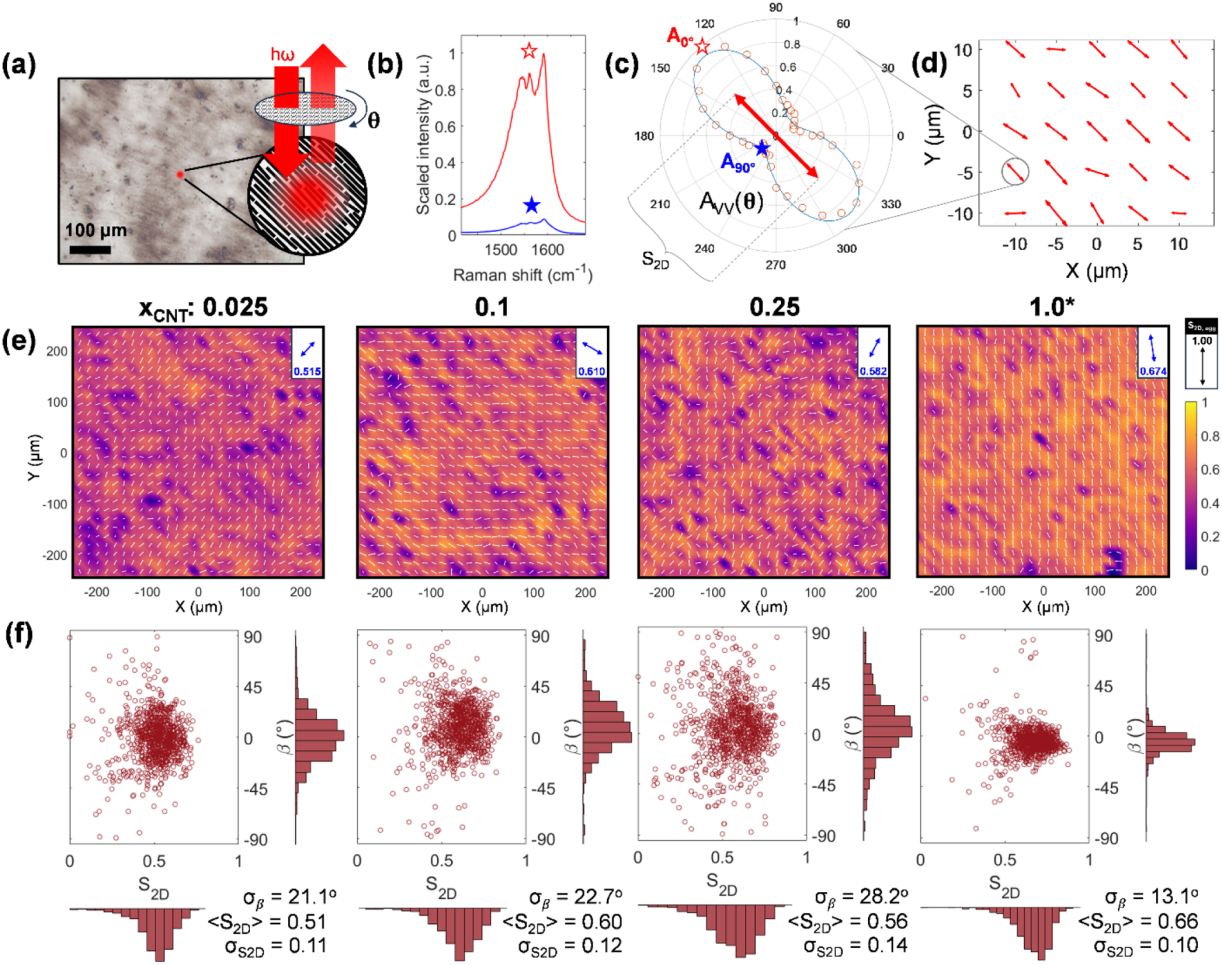
Detailed characterization of nematic textures in aligned BNNT/SWCNT composites, using SWCNTs as spectroscopic probes. (a) schematic of the light path of the incoming excitation and collected Raman scattering traveling through a rotatable waveplate to enable mapping of orientationally specific Raman scattering. (b) Representative polarized Raman spectra (λ_exc_ = 633 nm) of a metallic SWCNT-containing film at VV (0°) and HH (90°) positions. (c) Angle-dependent Raman scattering response quantified by peak area A_vv_(⊖), (d) converted to a vector the magnitude of which is the local orientational order parameter, S_2D_, forming part of an idealized vector field mapped across a film surface. (e) Vector fields of composites with varying SWCNT mass fraction (x_CNT_) across a 500 × 500 μm square section of each sample, in roughly 12-μm steps. Heat map intensity reflects the degree of coalignment of each point with the aggregate global director (blue double-headed arrow, top right inset, with aggregated macroscopic order parameter, S_2D,agg_). (f) Aggregate alignment statistics (S_2D_) and relative local orientations (β) collected from each individual probed sample point (*N* = 900 per panel).

In this work, we apply instead an advanced version of PRSM, differentiated by the polarization control being automated and implemented through placing a half-wave plate just before the microscope objective.^48^ These changes enable automated RS measurements at distinct sample orientations with respect to the incident laser polarization at each point; an example of this larger set of Raman scattering intensity versus orientation is shown in Figure 6c. This additional data in turn makes it possible to integrate and azimuthally resolve the SWCNT G-band mode intensity, and fit the angular response to generate a local, absolute, S_2D_ value.^11,14,35^

Applying the advanced PRSM method and making measurements across a grid of positions yields a vector field (depicted by red arrows in Figure 6d) combining the information on the local director orientation and its scalar magnitude of alignment (*i.e*., the S_2D_ value) at each position. This is a richer, multiscale, 2-D representation of the structural and nematic information that likens to the Oseen-Frank formalism used in treating liquid crystalline systems.^50^ A downside of collecting data in this manner is an increased acquisition time, as a sufficient number of angular RS collections must be conducted, and a lower number of acquisitions trades off certainty in the maximum local S_2D_ for determination of the mesoscale alignment direction (see Methods). Using all the polarization angle resolved data, i.e., the data of Figure 6c, for the same point as the orthogonal spectra of Figure 6b yields an S_2D_ value of 0.63, correcting for the absence of a VH intensity in the above S_2D_ calculation. For simplicity, the macroscopic alignment metric, S_2D,agg_, is closely related to the average over all grid points, < S_2D_ >; (full details in Methods below). To capture heterogeneities within and across neighboring nematic domains, gridpoint resolution and extent were chosen to probe across a large range of length scales, namely (2 – 15) μm and (40 – 500 μm), respectively. In Figure 6e we focus on the alignment maps acquired over the largest probed sample areas (with other scales featured in Figure S5).

The heat maps of Figure 6e were acquired on the same BNNT-SWCNT composite films containing only (2.5 – 25) % SWCNTs by mass characterized in Figure 5. These maps provide a multiscale summary of the salient structural features over 0.5 × 0.5 mm^2^ areas of each composite film: vector fields depicted as determined in Figure 6d are overlaid on a heat-map of the dot-product of each individual vector with the aggregate director field. This simultaneously allows probing of the alignment and shows the utility of SWCNTs to provide sufficient polarization-dependent signal even at sparse loadings. Results from a globally aligned SWCNT film (labeled as x_CNT_ = 1.0*, asterisk denoting that this sample was not of the same lot) are also featured as an alignment reference depicting an optimized SVF process with SWCNTs. The inset at the top right corner of each plot reports the global director orientation, as well as its magnitude (the aggregate macroscopic S_2D_ value, S_2D,agg_) for each scanned area and a reference vertical arrow of S_2D_ = 1. Finally, plotted in panel 6f are all mesoscopic orientations relative to the average global director against the local S_2D_ value at each location. Step size (i.e., map resolution) was selected to span a trade-off of throughput time and minimum domain size (which was observed to be ≈ 10 μm in neat BNNT films) such that a single film may be measured over 4 to 8 h.

Notably, with such maps, we are now able to discern competing mesoscopic effects and reconcile these with previously observed, compositionally varying nematic textures. For example, while the mean local S_2D_ (0.51 to 0.6) moderately increased with SWCNT fraction from 0.025 to 0.25, the S_2D_ variance and mosaic spread also increased, detracting from directional alignment on both macro- and microscopic scales. Given the inherent diameter and length mismatch of the BNNT and SWCNT nanotubes, it is not surprising that enriching the minority nanotube fraction would disrupt correlated order. These trends are also quite evident in the progressively more diffuse scatter of the computed local S_2D_ and orientational information as depicted in Figure 6f. Nonetheless, in the low-SWCNT limit, the overall alignment state over these macroscopic areas was fairly constant and similarly consistent with the lower S_2D_ bounds reported via LD. This leads us to conclude that films of BNNTs are highly amenable to alignment, and in addition to neat films can enable the creation of cocomposites with hosted SWCNTs and other anisotropic entities, to address a multitude of application venues.

## Discussion/Conclusion

We have surveyed the feasibility of global BNNT alignment by automated SVF. Alignment extent was mediated by nanotube length in a similar fashion to SWCNTs, resulting in significant ordering with either nanotube type. Achieving this ordering with broad length partitions sets less stringent tolerances on the length dispersity required and highlights the importance of excluding gross material impurities and short nanotubes from nanotube dispersions. Further, mixed composites showed promising ability to coalign. We report the characterization of neat BNNT alignment, in turn interrogating impacts of assembly parameters and multiscale structure of aligned nanotube thin film composites. Being bidisperse rodlike systems, BNNT/CNT composites were accordingly shown by our Raman-based methods to exhibit more diverse phase behavior and disorder relative to neat CNTs, nonetheless showing promising ability to coalign. Our observations set an elevated lower bound for the ordering achievable with SVF-based methods; improvements by surface modifications,^13,51^ greater material refinement,^26,37^ and a more detailed mechanistic understanding of assembly will further advance state-of-the-art fidelity. A particular next step of importance is likely adding diameter control^26^ to the BNNT length-separated populations to improve SVF optimization and uniformity in potential geometric packing. The material processing and metrological methods detailed herein position the field to more usefully apply assembled nanotube materials toward quantum-photonic, plasmonic, electronic, and structural applications.

## Methods

Certain equipment, instruments, software, or materials, commercial or noncommercial, are identified in this paper in order to specify the experimental procedure adequately. Such identification is not intended to imply recommendation or endorsement of any product or service by the National Institute of Standards and Technology (NIST), nor is it intended to imply that the materials or equipment identified are necessarily the best available for the purpose.

### Dispersion Preparation

Length sorting for nanotubes was conducted as described in Shapturenka et al.^37^ Parent arc-discharge SWCNT soot and dry synthesized BNNTs also originated from the same respective material lots (Carbon Solutions, P2 grade lot A011, and BNNT, LLC, batch BNNT B, Lot Y5B01220211B). Concentrated nanotube suspensions were bath-sonicated for 30 s prior to dilution. All aqueous parent nanotube dispersions were uniformly stabilized with 10.0 g/L sodium deoxycholate (DOC, Sigma-Aldrich BioXtra) in H_2_O. Parent dispersions were diluted to produce the working dispersions, typically 3 mL of 0.3 g/L aqueous DOC with either 4 or 8 μg of nanotube mass.

### Preparation of Nanotube Films

Filtrations were conducted through Whatman Nuclepore polycarbonate, polyvinylpyrrolidone-coated, track-etched membranes (80 nm pore diameter) in a Millipore filtration assembly connected to house vacuum and a PID-controlled parallelized pressure line via a digitally driven solenoid bleed-off valve. Applied pressures were in the range of 2 to 5000 Pa below atmospheric pressure. Permeate flux was calculated from a trace of meniscus height measured over time, tracked by machine vision of the dispersion fluid column against a uniform background. Filtration recipes were run within 10% of target flow rate, taking membrane and specific cake resistances into account with a programmed pressure ramp determined via Darcy’s law. More details can be found in the Supporting Information and Walker et al.^11^

### Film Transfer

Filtered films were transferred to glass coverslips for polarized microscopy and absorbance measurements, and to silicon wafer pieces for Raman scattering. Once dried, membranes were adhered to substrate surfaces with water, film side down, and left to dry completely. Chloroform was progressively introduced dropwise on top of the adhered membranes to prevent membrane buckling and detachment, then rinsed further and soaked in a chloroform bath for 10 min. These were then allowed to air-dry, washed in ethanol and water, and dried with pressurized air.

### Polarized Optical Microscopy (POM)

POM of transferred films was conducted on an Olympus BX51 microscope in a static cross-polarized configuration, with rotation stage position determining the relative film-polarizer orientation. Maximum I_BR_ was calculated as the mean pixel intensity from the sample/stage orientation yielding the brightest POM image. Imaging conditions (constant camera exposure time, brightness/contrast values, and zero gain) were consistent within each length-, composition-, and flux-dependent series, and were varied among different sample series to maximize the total dynamic range of brightness values within the RGB channel space. Birefringent contrast ( Δ BR) was calculated as 1 – I_BR,45°_/I_BR,0°_, with 0° arbitrarily denoting an azimuthal sample position yielding the brightest birefringence, and a rotation of 45° away from that position, respectively).

### UV–Vis–NIR Spectroscopy

Polarized absorbance was measured with a PerkinElmer Lambda 950 in a transmission geometry, with a Glan-Taylor polarizer crystal in a motorized rotation mount, filtering a deuterium-halogen UV–vis-NIR illumination source (≈ 3 × 1.5 mm diameter spot size). The detection range was (350 – 2000) nm.

### Raman Characterization

Raman spectroscopy was carried out on a Horiba LabRAM Evolution instrument with a helium–neon gas laser excitation at 632.8 nm (≈ 10 mW) and at 100× magnification. PRSM was carried out with a half-wave plate housed in a motorized rotation mount placed just above the microscope objective, allowing a variation in excitation laser polarization (θ) incident on the nanotube film surface without physically moving the sample. The Raman modes comprising the G-band were fit as Lorentzian peaks, integrated, and summed to comprise the angle-resolved signal profile at each point of the spatial mapping. This profile was then fit to the Raman-tensor derived expression relating the state transformation of the incident excitation to the scattering intensity; three empirical coefficients emerge that relate to the nematic order parameter, S_2D_:

3 $$A_{VV}\propto A{\;\cos}^{4}\theta +B{\;\cos}^{2}\theta {\;\sin}^{2}\theta +C{\;\sin}^{4}\theta$$

where A = $\left\langle \cos^{4}\beta \right\rangle$ , B = $3\langle \cos^{2}\beta \sin^{2}\beta \rangle$ , and C = $\frac{3}{8}\langle \sin^{4}\beta \rangle$ , with β the separation angle between the director and a given nanotube axis.^35^ These coefficients capture the distribution-dependent anisotropy of the resulting scattering response, assuming a Gaussian distribution of in-plane nanotube orientations at the nanoscale:

4 $$P\left(\beta \right)=\frac{1}{\sqrt{2\pi \sigma^{2}}}e^{-\beta^{2}/2\sigma^{2}}$$

σ was numerically obtained by minimizing mean-squared error between the generated angle distribution and the distribution captured by the ABC coefficients and converted to a local S_2D_ parameter. The aggregate nematic order parameter, S_2D,agg_, was calculated by summing all distributions, renormalizing, and integrating over the resulting distribution.

## Supplementary Material



## Notes

Atomic force micrographs of coarse centrifugation refinement, absorbance spectra of parent material, coarsely refined material, and assembled nanotube films, details of flux-dependent SWCNT assembly behavior, further SEM imaging and Raman spectroscopy of nanotube films, lengths of polymer-sorted nanotube samples, detailed description of SVF analysis via Darcy’s Law (PDF)
