# Supplementary material for: Aligned Boron Nitride Nanotube Thin Films and Their Cocomposites with Single-Wall Carbon Nanotubes through Slow Vacuum Filtration
Source: ACS Nanosci Au. 2025 Jun 17;5(4):293–305. doi: 10.1021/acsnanoscienceau.5c00022 (PMC12371585; doi:10.1021/acsnanoscienceau.5c00022)
Supplement: Supplementary file 1 [file ng5c00022_si_001.pdf]

Supporting Information for

Aligned Boron Nitride Nanotube Thin Films and their Co-Composites with Single Wall Carbon Nanotubes through Slow Vacuum Filtration

Pavel Shapturenka, Tehseen Adel, Frank M. Abel, Angela R. Hight Walker, and Jeffrey A. Fagan*

*Corresponding author. Email: Jeffrey.fagan@nist.gov

Official contribution of the National Institute of Standards and Technology
Not subject to copyright in the United States

Certain equipment, instruments, software, or materials, commercial or non-commercial, are identified in this paper in order to specify the experimental procedure adequately. Such identification is not intended to imply recommendation or endorsement of any product or service by the National Institute of Standards and Technology (NIST), nor is it intended to imply that the materials or equipment identified are necessarily the best available for the purpose.

This PDF file includes:

Figs. S1 to S6
Table S1
Appendix: Specific cake resistance determination

Acronyms:

Analytical ultracentrifugation (AUC)
Arbitrary unit (a.u.)
Atomic force microscopy (AFM)
Birefringence (BR)
Birefringent contrast (ΔBR)
Birefringence intensity (I_{BR})
Boron nitride nanotube (BNNT)
Electric arc method synthesized nanotubes (EA)
Linear dichroism (LD)
Near infrared (NIR)
Polarized optical microscopy (POM)
Polymer depletion length separation (PDLS)
Polarized Raman scattering microscopy (PRSM)
Raman Scattering (RS)
Rate-zonal (RZ) ultracentrifugation sorting
Single-wall carbon nanotube (SWCNT)
Slow vacuum filtration (SVF)
Sodium cholate (SC)
Sodium deoxycholate (DOC)
Ultraviolet (UV)
UV-visible-near infrared (UV-vis-NIR)

1. AFM Characterization of BNNT Populations with Different Degrees of Centrifugation

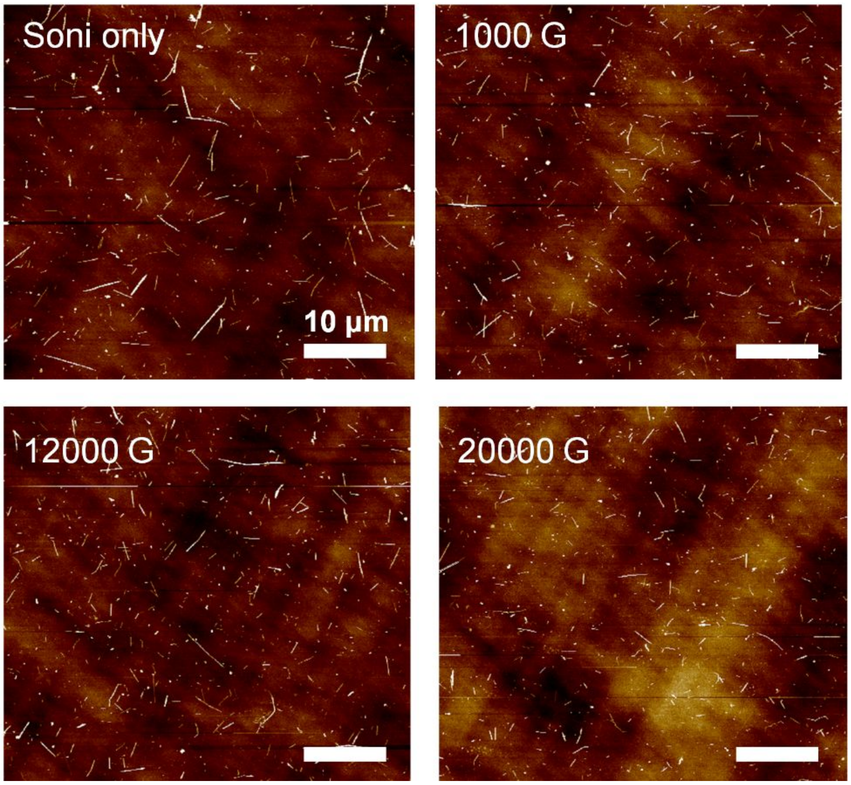

Figure S1. AFM images of supernatant from BNNT dispersions centrifuged at various speeds. In line with the measured object lengths reported in the main text, these images suggest that centrifugation removed large outliers and gross morphological impurities preventing high values of cake resistance in SVF. Here, “G” stands for the gravitational acceleration constant (9.81 m/s^2).

2. Optical Characterization of Parent Nanotube Populations and LD of films

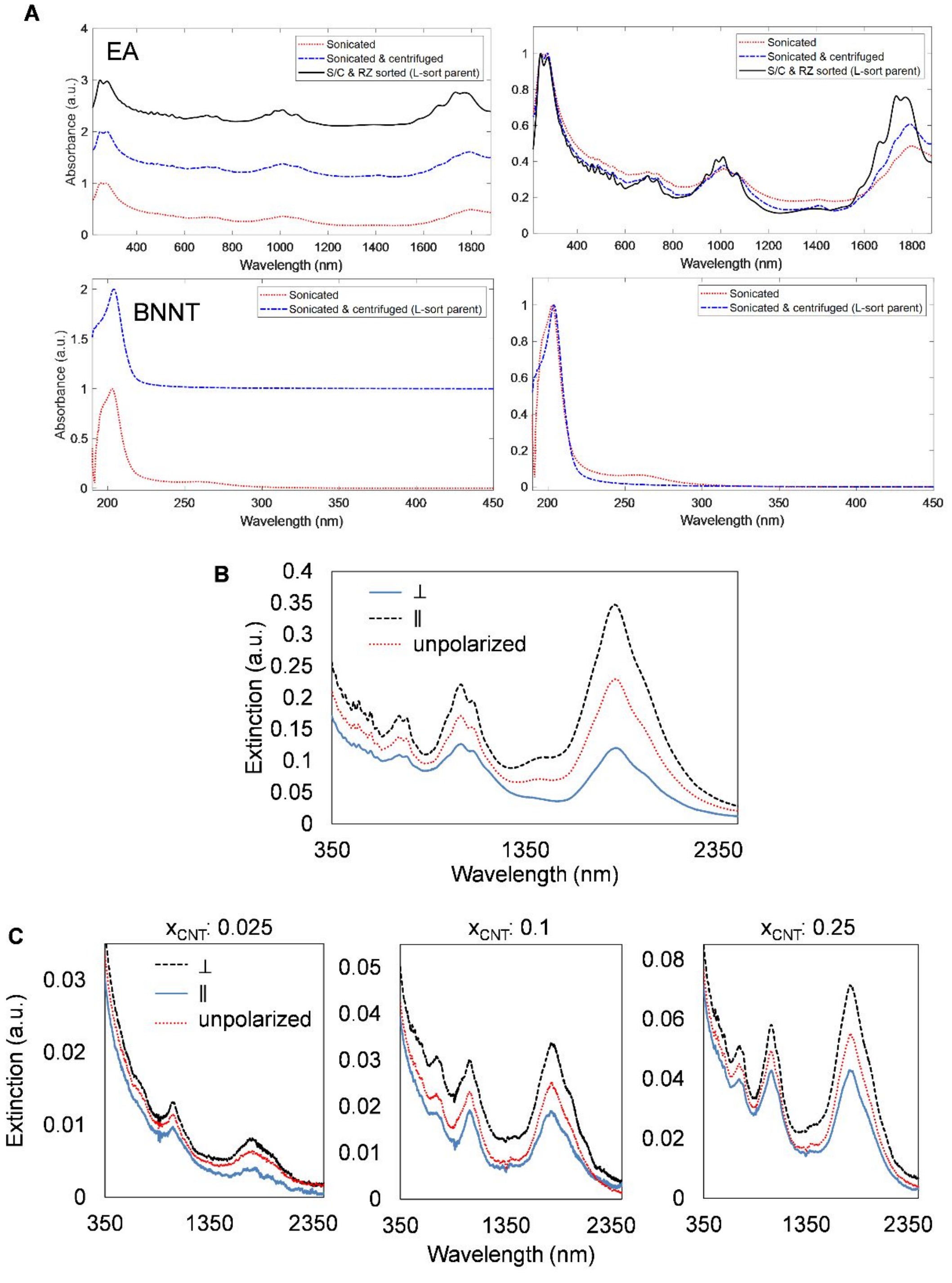

Figure S2. Optical properties of parent material solutions and solid films.

(A) Scaled (right) and scaled and offset (left) UV-vis-NIR absorbance spectra curves for aqueous dispersions of EA SWCNTs and BNNTs. Reproduced from Shapturenka, P. *et al., RSC Adv.* **2024**, 14 (35), 25490–25506 with permission from the Royal Society of Chemistry. (B) Polarized UV-Vis-

NIR spectra of a film assembled from the A5 SWCNT fraction *via* SVF oriented parallel and perpendicular to the incident light polarizer, as well as an unpolarized spectrum for reference. (C) Polarized UV-Vis-NIR spectra of BNNT/SWCNT composites at various CNT/BNNT ratios. The increase in extinction at shorter wavelengths is attributable to light scattering; note that the short wavelength limit of our Glan-Thompson polarizer is ≈ 340 nm, so measuring linear dichroism of all-BNNT samples was not possible.

3. Birefringence Characterization of A4 SWCNT SVF Films

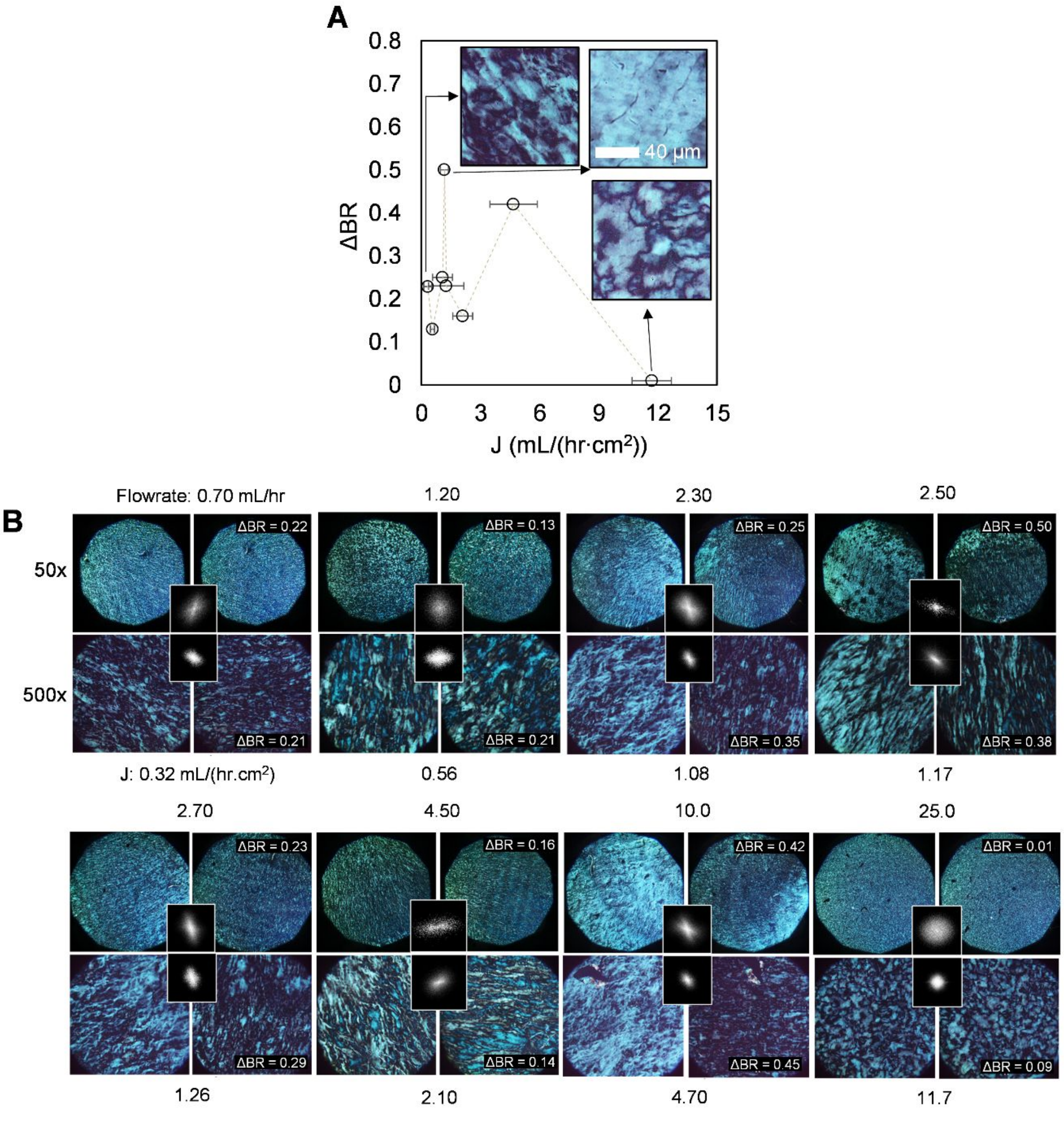

Figure S3. Effect of filtration flux on the microstructure of thin films comprised of length-sorted SWCNTs (A4 fraction, average length ≈ 540 nm).

(A) Measured birefringence contrast (ΔBR) of SVF-assembled EA SWCNT films across a variation in filtration flux, J. (B) POM images of all samples included in the flux series at various

magnifications, along with their fast Fourier transforms and ΔBR values. Note that the labelled values on top of each subpanel are flowrate (mL/hr), and the values below are in units of flux (mL/(hr cm^2)).

4. Birefringence Characterization of an unsorted SWCNT Parent and "Excluded" SVF Films

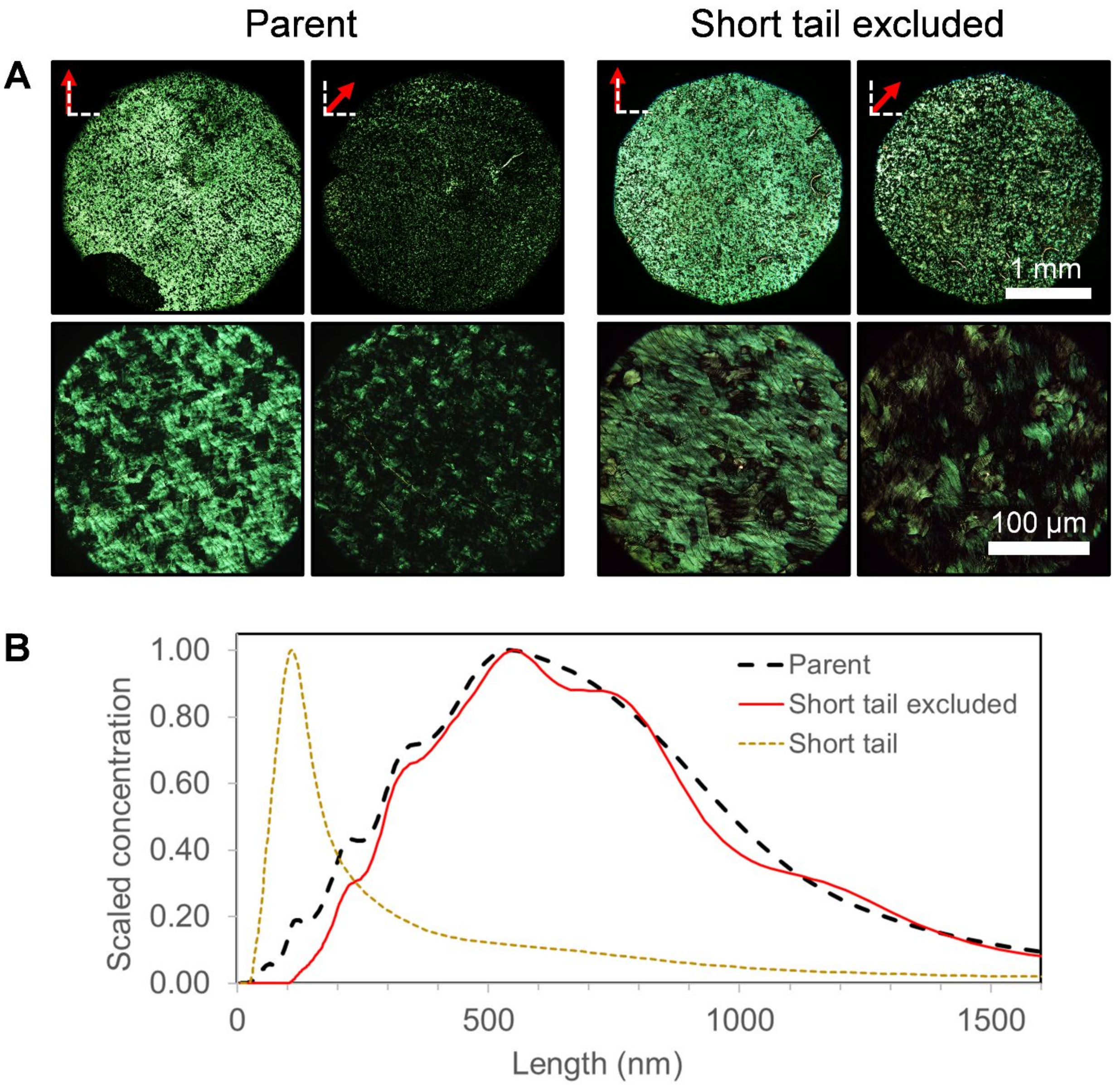

Figure S4. Effect of removing short nanotubes from EA SWCNT dispersions filtered by SVF.

(A) Polarized optical microscopy of thin SWCNT films formed from a purified, non-length sorted dispersion (left) and the same dispersion with the shortest nanotubes removed from the population (right). Domain sizes visibly enlarge, elongate, and become more uniform with short tail removal. Note that the absolute contrast and other camera imaging settings were not the

same for these two sets of images, such that we cannot make a meaningful comparison of the two using birefringence metrics. (B) Length distributions (signal weighted ≈ mass weighted) of the parent, short-excluded, and residual (short tail) nanotube populations as measured by AUC. Note that the mass concentration of such short nanotubes is quite modest (as demonstrated by the overlaid parent and exclusion-sorted traces), but significantly impacts the long-range structural arrangement of resulting thin films.

5. **Example PRSM Orientation Maps**

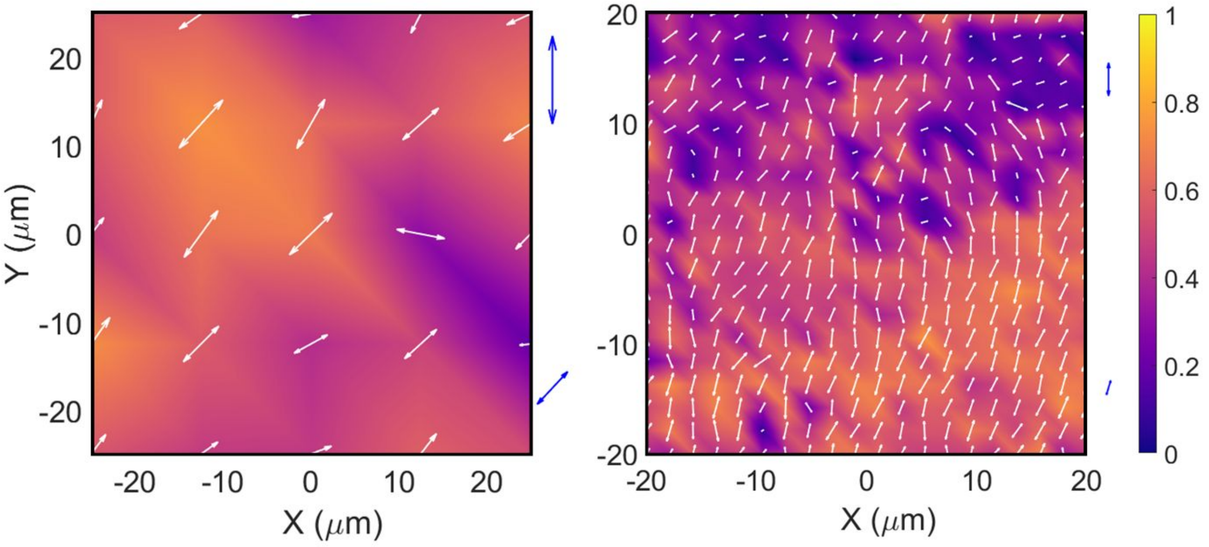

Figure S5. Multiscale orientational state maps of SWCNTs within BNNT/SWCNT thin film composites (x_{CNT} = 0.25) probed by PRSM.

(Left) low resolution, (Right) greater spatial resolution PRSM maps of the local alignment director and magnitude (arrow length). Vectors at all grid points represent the absolute orientation of the probed nanotube ensemble, while their magnitude is the 2-dimensional nematic order parameter, S_{2D}. The blue arrow at the top right of each map is a vertical reference arrow with a magnitude of unity, and the arrow at the lower right reflects the average orientational state of all probed points in the grid (nominally the aggregate director). The heat map depicts an S_{2D}-weighted dot product between the aggregate director and each individual point.

In contrast to the macroscopic maps featured in the main text, the gridpoint resolution is high enough to resolve neighboring domains across the entire scanned area, which is particularly visible in the right panel. A gradual variation in point-to-point orientation highlights areas within

a single domain, whereas adjacent regions adopting different local directors implicitly delineate nematic grain boundaries.

6. **Electron Microscopy of BNNT films**

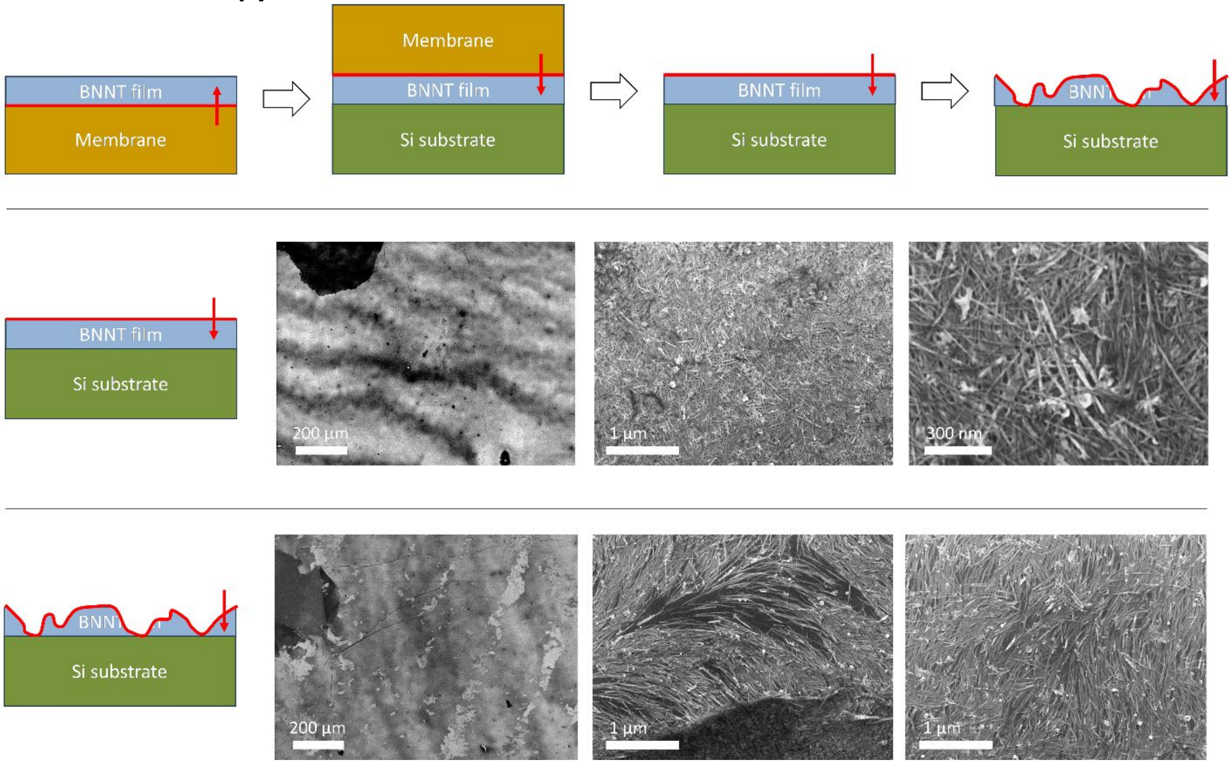

Figure S6. Uncovering liquid crystalline order in BNNT films with electron microscopy. Scanning electron microscopy of BNNT films exhibiting the greatest observed global alignment provided several insights to suggest that the underlying BNNT ordering mechanisms during SVF are distinct from those observed for SWCNTs in prior work. Namely, imaging the top surface of transferred membranes (the nanotubes in immediate contact with the filtration membrane) shows a highly random BNNT network. Exfoliating this topmost layer uncovered a correlated nematic domain texture with common directors exceeding 10 µm in radius. Contrary to what is hypothesized for SWCNT alignment, images suggest the first BNNT layer was arrested almost immediately on the membrane in a random network, perhaps due to significant membrane-nanotube adhesion forces. Thereafter, the comparatively weaker interaction between the formed BNNT monolayer and subsequent descending nanotubes would allow nematic domains to form and influence the ordering dynamics of surrounding neighbors.

Table S1.

Number- and mass-averaged lengths (<L_M> and <L_N>, respectively) of all nanotube fractions used for SVF-based film formation. Data reproduced from Shapturenka, P. *et al., RSC Adv.* **2024**, 14 (35), 25490–25506 with permission from the Royal Society of Chemistry.

	Fraction	<L_N> (nm)	<L_M> (nm)
**EA SWCNT**	**A5**	619.8	752.2
	**A4**	453.7	536.8
	**A3**	246.7	295.8
	**A2**	164.2	194.1
	**A1**	107.6	133.8
**BNNT**	**B5**	500.9	716
	**B4**	622.2	719.8
	**B3**	409.4	453.7
	**B2**	172.5	203.4
	**B1**	137.4	169.9

Appendix: Specific cake resistance determination

Specific cake resistance, α_{cake}, was determined using two distinct methods of Darcy's law analysis. To re-iterate the definition of Darcy's Law from the main text, the law relates transmembrane pressure gradient, ΔP, and time-dependent permeate flux, J(t):

$$\frac{1}{J(t)} = \frac{\mu(R_m + \alpha_{cake} C v(t))}{\Delta P} \quad [1]$$

in which μ is the filtrate viscosity, R_m is membrane resistance, C is the initial nanoparticle dispersion concentration, and v(t) is the time-dependent, area-normalized permeate volume.
ΔP is a known and controlled quantity, given by a sum of applied vacuum pressure and hydrostatic pressure of the filtrate column.

The first α determination method can be depicted by plotting the inverse flux against filtration progress as measured by total permeate volume (normalized by area to yield a "meniscus travel distance", v), which tends to linearity as the filtration progresses. The absolute value of the slope of this linear regime ($\frac{1}{C(t)v}\left(\frac{\Delta P}{\mu J(t)}\right)$) yields α_{cake}:

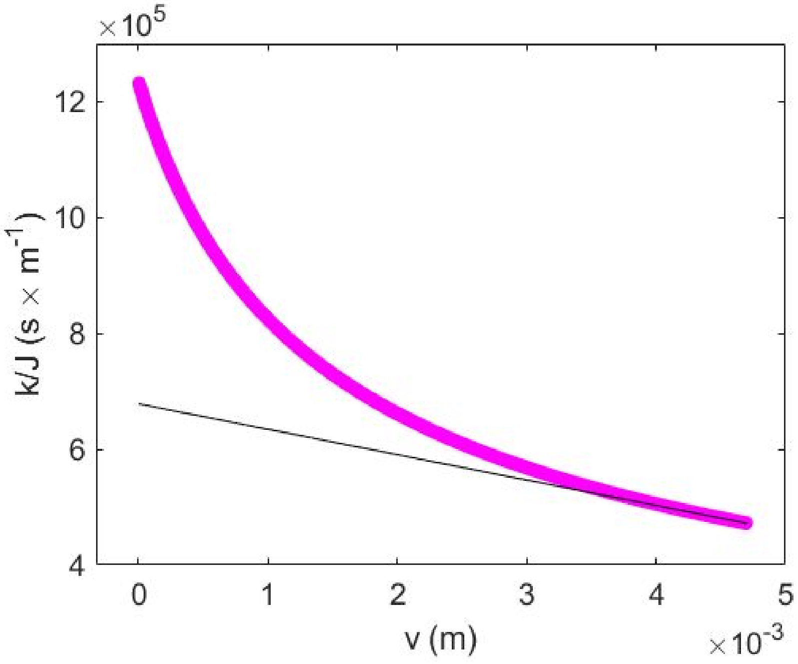

Figure S7. Graphical Presentation of the Asymptotic Slope Evaluation of α_{cake}

The inverse of the permeate flux decreases as the meniscus travels (towards the filter) and collects material in the cake layer on the membrane. Eventually the slope approaches the value for α_{cake}. Note that in SVF for aligned membrane production the pressure differential across is controlled to instead set a constant permeate flux.

In the alternative method, we bin the instantaneous α calculated at each point in time:

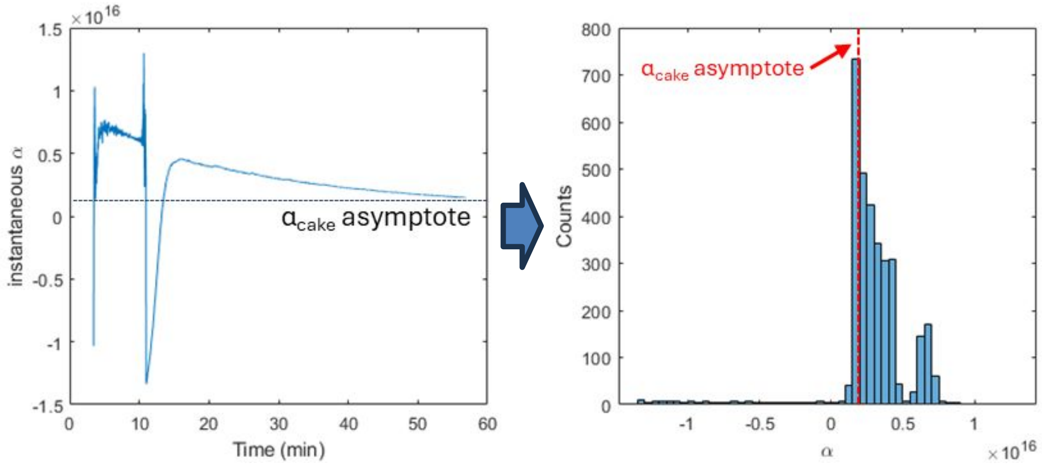

Figure S8. Alternative use of Instantaneous Values for Determining α_{cake}

A plot of an example determined instantaneous α_{cake} values as a function of time. α_{cake} trends to the material-determined value as filtration progresses and becomes more dominated by the cake resistance.

While the use of the instantaneous α_{cake} representation yields individually less robust values, the resulting distribution informs the physical range for these values over the course of filtration, acting as a helpful reference and point of redundancy. A convergence to a single cake resistance value is also evident in this implementation as the filtration progresses.
